# Kidney Transplantation: The Challenge of Human Leukocyte Antigen and Its Therapeutic Strategies

**DOI:** 10.1155/2018/5986740

**Published:** 2018-03-05

**Authors:** Tilahun Alelign, Momina M. Ahmed, Kidist Bobosha, Yewondwossen Tadesse, Rawleigh Howe, Beyene Petros

**Affiliations:** ^1^College of Natural Sciences, Department of Microbial, Cellular and Molecular Biology, Addis Ababa University, P.O. Box 1176, Addis Ababa, Ethiopia; ^2^Department of Biology, Debre Berhan University, P.O. Box 445, Debre Berhan, Ethiopia; ^3^Armauer Hansen Research Institute, Addis Ababa, Ethiopia; ^4^St. Paul's Hospital Millennium Medical College and Addis Ababa University, Addis Ababa, Ethiopia; ^5^School of Medicine, College of Health Sciences, Addis Ababa University, Addis Ababa, Ethiopia

## Abstract

Kidney transplantation remains the treatment of choice for end-stage renal failure. When the immune system of the recipient recognizes the transplanted kidney as a foreign object, graft rejection occurs. As part of the host immune defense mechanism, human leukocyte antigen (HLA) is a major challenge for graft rejection in transplantation therapy. The impact of HLA mismatches between the donor and the potential recipient prolongs the time for renal transplantation therapy, tethered to dialysis, latter reduces graft survival, and increases mortality. The formation of pretransplant alloantibodies against HLA class I and II molecules can be sensitized through exposures to blood transfusions, prior transplants, and pregnancy. These preformed HLA antibodies are associated with rejection in kidney transplantation. On the other hand, the development of de novo antibodies may increase the risk for acute and chronic rejections. Allograft rejection results from a complex interplay involving both the innate and the adaptive immune systems. Thus, further insights into the mechanisms of tissue rejection and the risk of HLA sensitization is crucial in developing new therapies that may blunt the immune system against transplanted organs. Therefore, the purpose of this review is to highlight facts about HLA and its sensitization, various mechanisms of allograft rejection, the current immunosuppressive approaches, and the directions for future therapy.

## 1. Introduction

### 1.1. Human Leukocyte Antigens (HLA)

The major histocompatibility complex (MHC) is a gene region coding for cell surface proteins important for the immune system. MHC is the most complex immunogenetic system presently known in humans. The human MHC is often referred to human leukocyte antigens (HLA), which is the name given for gene clusters. Although HLA are known as human leukocyte antigens, they mostly exist on the surfaces of our cells [1]. HLA are groups of cell surface proteins encoded by genes in MHC which are known as HLA in humans and H-2 in mice [2]. HLA genes are located on the short arm of chromosome 6 at 6p21 position [3, 4], occupying a genetic region of 4 Mbps [5]. The human immune system uses HLA's uniqueness to distinguish self from nonself. HLA are responsible for the presentation of “foreign” peptides (antigens) to the immune competent cells. T lymphocytes recognize foreign antigens only when it combines with HLA molecules.

The overall size of the HLA is approximately 3.6 million base pairs (~3.6 Mbp) [4] or about 0.1% of the human genome. Each class I and class II gene spreads over approximately one-third of the HLA length. About 224 gene loci were described out of 3.6 Mbp HLA complex super-locus [6]. HLA is the most gene-dense region of the human genome [5]. The HLA gene complex alone contributes more than 10% of genetic diversity in humans. Most of the allelic variations in the HLA genes are in exons 2 and 3 for class I and exon 2 for class II, which code for the antigen-binding regions of the proteins. Differences between different alleles are due to multiple single-nucleotide polymorphisms (SNPs), which suggests that the mechanism of allele formation is via segmental exchange of alleles at the same locus. In other words, there is a patchwork kind of sequence motif variation which could have arisen from recombination [7].

Based on the structure of the antigens produced and their function, there are two classes of HLA, HLA class I and class II. Some studies have clustered the genes into three separate loci, that is, HLA class I, class III, and class II [8]. Class I histocompatibility antigens (HLA-A, B, and C) are expressed on all cells, and class II histocompatibility antigens (HLA-DP, DQ, and DR) are expressed on antigen-presenting cells (B-cells, macrophages, dendritic cells, Langerhans cells, and capillary endothelium). Histocompatibility antigens are inherited from both parents as MHC haplotypes [9–11]. This is composed of 5 to 8 exons and ranges in length from 4 to 17 kb [4]. HLA includes several loci closely linked, and each of these loci involves numerous alleles, having 40 to 60 alleles per locus that control the production of their corresponding antigens [12]. HLA mismatches may occur at antigenic or allelic levels; the first are characterized by amino acid substitutions in both peptide-binding and T-cell recognition regions, whereas the latter are characterized only by amino acid substitutions in the peptide-binding regions [1].

The protective mechanisms of the human immune system use HLA molecules to bind peptide antigens and present fragments of antigens to T lymphocytes [9–11]. HLA is a gene complex whose alleles encode polymorphic cell surface glycoproteins which are involved in antigen recognition and presentation. HLA molecules are surface glycoproteins having a peptide-binding ability with their peptide-binding regions [13]. Depending on the genetic disparity between the donor and the recipient, graft and host HLA molecules present different peptides. HLA class I molecules are expressed on nucleated human cell surfaces and present non-self-antigens to cytolytic CD8 T-cells, and HLA class II molecules are expressed on antigen-presenting cells and present the antigen to CD4 T helper cells [1, 14, 15].

### 1.2. The Structure of Class Molecules

**Figure 1:**
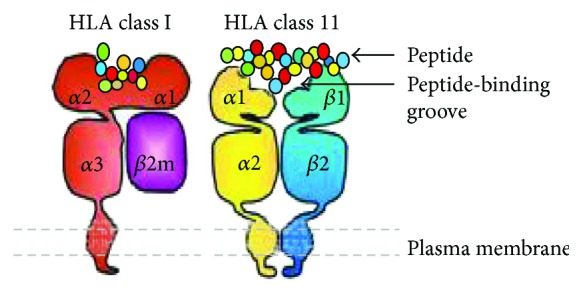
HLA classes I and II are heterodimeric transmembrane proteins (adopted from [19]).

MHC-I and MHC-II genetic regions of the MHC are interspersed by the MHC-III region between them but code for structurally different proteins [16]. Class I HLA consist of a polymorphic *α*-chains associated with a nonpolymorphic *β*2-microglobulin chain. These are widely distributed on nucleated cells and are particularly abundant on lymphoid cells and vascular endothelium [17]. Unlike class I molecules, HLA class II antigens are composed of two chains (*α* and *β*) encoded by two distinct genes (A and B) [18]. Class II HLA are covalently linked dimers of *α*- and *β*-chains. Class II antigens are expressed at high density by APCs [12]. Antigenic variability in class I is less than class II because class I is encoded by one less polymorphic locus of *β*2-chain [17]. HLA class I is made up of a heavy chain with three globular domains (*α*1, *α*2, and *α*3) noncovalently bound to *β*2m. HLA class II is made up of two heavy chains (*α*-chain and *β*2-chain) each with two globular domains (*α*1 and *α*2 or *β*1 and *β*2). The *α*1 and *α*2 domains of HLA class I and the *α*1 and *β*1 domains of HLA class II make up the peptide-binding groove as shown in Figure 1 [19].

Genes of class I (HLA-A and B) and class II (DRB1) are the most polymorphic loci across the HLA complex with 3830, 4647, 3382, and 2011 alleles, respectively [20]. Polymorphism means the occurrence of several alleles, that is, genes encoding various MHC antigens located at the same locus. Polymorphisms of HLA genes especially in the peptide-binding regions are functionally important as they may lead to variations in the peptide-binding abilities and specificities [7]. HLA genetic diversity/variation occurs at the population level [21]. Every individual has two alleles for every HLA, one inherited from each parent [22].

**Figure 2:**
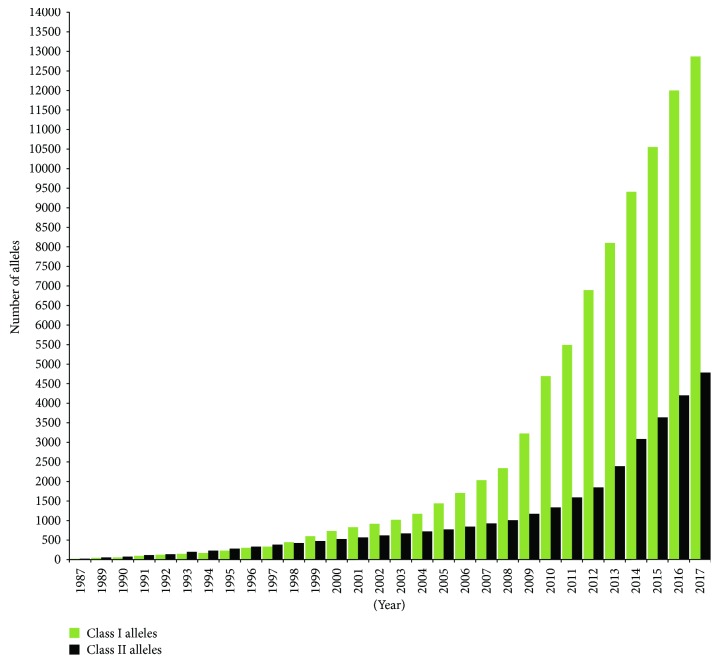
Graph showing the number of alleles named by year from 1987 to the end of December 2017 (adopted from [20]).

HLA alleles described by the IMGT/HLA database consist of 13,412 alleles, of which HLA-B have the highest number of alleles (3977) [21]. About 103 class I epitopes have been identified [23]. However, there is no complete list of alleles for each locus within the HLA system [7] because there are an increasing number of HLA alleles identified as genotyping technique increase (Figure 2). This implies the need for the development of new tools for the detection of newly identified HLA alleles implying that new drugs will be needed for the immunosuppression of these HLA alleles for successful transplantation [24]. In transplantation, the more closely the donor and the recipient alleles matched, the lesser the risk for tissue rejection [10]. Therefore, high resolution of HLA typing which is not restricted to the peptide-binding region can decrease HLA allele ambiguities [4, 25].

### 1.3. Allele Assignment and Nomenclature

**Figure 3:**
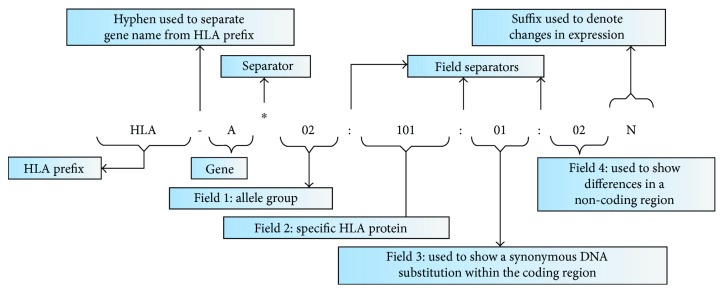
New HLA nomenclature patterns (adopted from [8, 20]).

HLA nomenclature differs depending on the type of method used. The initial alleles were defined by serological methods; however, the use of molecular methods has resulted in a more precise designation of HLA alleles [16]. Looking at the HLA at the DNA level [10, 17], HLA nomenclature is a new nomenclature that was introduced in 2010 [8]. This is because the old system could no longer accommodate the increasing number of HLA alleles. Currently, over 5700 alleles were described across the HLA loci. The DNA-based naming classification system is much accurate than the serological system [26]. In the HLA system, *haplotype* is the linkage of particular alleles at distant loci that occur as a group on a parental chromosome. It segregates as a linkage group from parents to children [16] (Figure 3).

### 1.4. HLA in Kidney Transplantation

Kidney transplant is the gold standard therapeutic strategy of replacing renal dysfunctions that offers the best survival to the patients with end-stage renal failure [22, 27, 28]. It is the transfer of living cells, tissues, or an organ from one individual to another (allograft). Kidney transplantation is associated with 68% lower risk of death than dialysis [29]. Kidney donors could be either deceased or living sources [30]. In 1954, the first successful kidney transplantation was performed between identical twins in Boston [22]. In Ethiopia, kidney transplantation has been performed on September 2015 at National Kidney Transplant Center under the auspice of St. Paul's Millennium Medical College with the Help of the University of Michigan [31].

The law of transplantation indicates that grafts between genetically identical individuals survive and grafts on genetically different individuals fail [32]. Usually, transplant rejection of the kidney occurs when the immune response of the recipient recognizes the new kidney as being a harmful object. This remains a major immunological barrier to organ transplantation therapy [10, 33, 34]. Thus, prior knowledge of the existing anti-HLA antibodies circulating among potential kidney recipients is important to set protective measures before transplantation.

### 1.5. HLA Sensitization

HLA sensitization refers to the presence of antibodies in the potential recipient against HLA molecules of the selected donor. Exposure to nonself HLA can cause the production of HLA-directed antibodies. Alloantibodies recognize antigenic epitopes displayed by the HLA molecule on the transplanted allograft and contribute to graft damage. There is a clear association between previous exposure to foreign HLA and the occurrence of a high degree of panel reactive antibody (PRA) [28]. The percentage of PRA estimates the likelihood of positive crossmatch to potential donors [35], and patients with greater quantities of preformed DSA have the highest likelihood of graft loss [36]. Donor-specific anti-HLA antibodies (DSA) identified by single-antigen bead (SAB) array are questioned for their sensitivity and lack of event prediction after transplantation. Despite known technical limitations of SAB assay [37], it appears to be a highly useful tool for posttransplant monitoring of HLA antibodies and surveillance of antibody-mediated rejection.

The impact of sensitization in a potential recipient results in longer waiting time for transplantation, posttransfusion complications, exposure to more adverse effects of immunosuppressor drugs, and finally graft rejection [38, 39]. The common causes of HLA-sensitizing events include previous transplants, pregnancies, and blood transfusions that lead to the development of DSA [15, 40–42]. The risk of sensitization increases as there is exposure to more than one sensitizing factor [28].

#### 1.5.1. Previous Transplants

Anti-HLA antibodies produced after kidney transplantation are risks for transplant survival [43–45]. After posttransplant, 24% of renal allograft recipients will develop de novo HLA-DSA within ten years [46]. Retransplantation showed stronger antibody production than the first exposure of transplantation [40] and increases the risk of early graft loss [47]. Patients would be broadly sensitized after the removal of the failed renal allograft [48]. Multiparous women who have lost their previous grafts are the highest risk factors of organ rejection [49]. Studies demonstrated that previous organ transplantation, pregnancies, and transfusions are common HLA-sensitizing factors [40, 44, 49]. The disparity of HLA of class I (HLA-A and B) [50, 51], HLA-C [52, 53], and class II (HLA-DR) [50, 51] between a recipient and a donor is a major antigenic barrier to transplantation therapy. Thus, minimizing HLA mismatch between a donor and a recipient is required to ensure successful transplantation [54–56].

#### 1.5.2. Pregnancy

In women, pregnancy remains an unavoidable HLA-sensitizing event [48]. Sensitization by pregnancy has a significant impact on the development of HLA class I and class II antibodies. The rate of developing HLA-B antibodies in patients sensitized by pregnancy was higher compared with sensitization after transfusion [57]. Studies showed that the risk of large increases in donor-specific antibody was greatest when antibodies were originally stimulated by pregnancy than transplant antigens [58]. Similarly, it has been also reported that the prevalence of anti-HLA antibodies was higher in pregnant women than transplant and transfusion events [57].

A baby inherits its HLA type from each parent, and the mother is exposed to the father's antigens which are expressed in the cells of the developing baby. The HLA from the father are foreign to the mother's immune system. HLA antibodies made during pregnancy do not cross the placenta and harm the baby. Antibodies to HLA class I are more frequent than class II [19]. The anti-HLA antibody development in pregnancy appears to be associated with the expression of particular HLA alleles [44]. In females, multiple pregnancies expose them to develop anti-HLA antibodies to the fetal antigens of a paternal origin which prevent them from being potential blood donors or recipients [59, 60]. The prevalence of HLA antibodies increases as the number of pregnancies/parity increases [61]. The direct sensitization of a woman against her partner and/or child makes them unsuitable potential donors for the mother [35]. Similarly, a study indicated that female patients receiving kidney allografts from their male partners or offsprings experience higher rates of graft rejection [62].

#### 1.5.3. Transfusion

The ABO system antigens are the most important blood cell antigens in transfusion. These antigens are complex carbohydrates (polysaccharides) expressed on the surface of RBCs and many other cell types such as vascular endothelium. Despite proper ABO antigen crossmatching, patients would experience transfusion reactions when they receive multiple blood supply [63]. ABO-incompatible organ transplant can cause hyperacute rejection due to the presence of preformed hemagglutinin A and/or B antibodies to nonself A or B antigens [35].

In blood transfusion, the leading cause of mortality is transfusion-related acute lung injury (TRALI) [64, 65]. Antibodies in the donor's blood could activate the recipient's pulmonary neutrophils, which damage the pulmonary endothelium resulting in pulmonary edema [66]. Transfusion is poorly immunogenic [62], and multiple transfusions are required to induce persistent HLA allosensitization [67]. The use of blood transfusions that matched for HLA-DR antigens was the starting point in transfusion therapy [68]. Platelets express HLA, but not red blood cells. The use of HLA-matched blood [61, 69] and leukocyte-depleted blood products [70] reduce the risk of HLA sensitization [67].

### 1.6. Non-HLA Antibodies and Acute Rejection

In the absence of circulating donor-specific HLA antibodies, non-HLA antibodies were shown to cause graft rejection [71]. The antigenic targets of non-HLA antibodies may be minor histocompatibility antigens, vascular receptors, adhesion molecules, and intermediate filaments [8]. The major histocompatibility class I-related chain genes are non-HLA proteins with some homology to HLA class I molecules and are frequent targets of the alloimmune humoral responses [72, 73]. Different non-HLA antibody types were identified as antiendothelial monocyte, antiactivated endothelial cells, or antiepithelial cells among patients who have rapidly rejected multiple renal allografts [74].

Non-DSA functions as complement and noncomplement-fixing antibodies which induce acute and chronic allograft injuries [8]. Non-HLA antibodies can be directed against polymorphic or nonallelic proteins. Antibody development against nonpolymorphic targets results from inflammations that break humoral tolerance to autoantigens [71]. Moreover, HLA genes are risk factors for most autoinflammatory diseases, which predispose humoral responses against self-antigens [75]. On the other hand, exposure to pathogens (such as bacterial and viral antigens) may trigger cross-sensitization and allograft rejection by enhancing the immune response to allogeneic HLA [39, 76]. Therefore, the effect of non-HLA antibodies on allograft rejection is a complex matter and the mechanisms of graft injury remain unclear.

## 2. Mechanisms of Graft Rejection

Rejection is defined as an immune response that mediates injury and destruction of transplanted tissues. Kidney transplant rejection is a complex process [77], and the graft could be viewed as a one-way process in which host immune cells destroy a defenseless allograft [78]. HLA molecules expressed on the surface of the donor cells induce an antigenic stimulus recognized by the recipient's immune system which triggers graft rejection [79]. The immune response to an allograft rejection involves both the innate and the acquired immune systems. The innate immune system predominates in the early phase of response through recognizing host-derived molecules which results from tissue damage. Proinflammatory damage-associated molecular patterns, hypoxia-inducible factors, adhesion molecules, dysfunction of the renal vascular endothelium, chemokines, cytokines, and Toll-like receptors are involved in the activation and recruitment of immune cells into injured kidneys. Immune cells of both the innate and the adaptive immune systems such as neutrophils, dendritic cells, macrophages, and lymphocytes contribute to the pathogenesis of renal injury [80]. Initiated inflammatory events by chemokines and cell adhesion molecules play essential roles, not only for leukocyte migration into the graft but also for facilitating dendritic cells and T-cells trafficking between lymph nodes and transplants [81]. The mechanisms of allograft rejection mainly depend on the adaptive immune system mediated by a complex interplay of cellular and humoral immunity [82, 83].

### 2.1. Cellular Graft Rejection

T-cells are critical in graft rejection due to TCR-MHC interactions and are mainly responsible for chronic rejection of most tissues [2]. T lymphocytes can directly recognize and respond to foreign HLA molecules. Subsequently, T-cells and cells of innate immunity function synergistically to reject the allograft [81]. Toll-like receptors and the complement systems are well-characterized components of innate immunity which are central to graft injury [84]. Pattern recognition receptors (PRRs) are a family of TLRs that are expressed on the outer cell membrane especially on APCs such as dendritic cells and macrophages. They have the ability to recognize pathogen-associated molecular patterns (PAMPs) to elicit innate immunity [85]. The ligation of APCs with antigens initiates intracellular signal transduction cascades that lead to nuclear factor-kappa B (NF-*κ*B) activation and the upregulation of the adhesion molecules, costimulatory molecules, and cytokines that are essential to immune activation. NF-*κ*B is a protein complex that controls the transcription of DNA, cytokine production, and cell survival [85, 86].

#### 2.1.1. Toll-Like Receptors

Toll-like receptors (TLRs) are pattern recognition receptors (PRRs) that sense tissue damage-associated molecular patterns (DAMPS) through their endogenous ligands [86]. This activates DCs [32], to secrete proinflammatory cytokines, upregulate surface MHC class II, and increase expression of T-cell costimulatory molecules, and it uses specific chemokine receptors that facilitate their trafficking to secondary lymphoid organs promoting acute rejection. Unlike DCs, macrophages probably do not play a direct role in the induction of allorecognition because they inefficiently prime naive T-cells [83]. Infection and cell injury result in the production of PAMPs and DAMPs that promote inflammatory responses via TLRs located on the cell membrane and within endosomes [86].

#### 2.1.2. Complement

The small peptide cleavage fragments of the complement system, C3a and C5a, are chemoattractants which interact with receptors on cells promoting acute graft rejection [32]. Complement receptors (CR) are PRRs that sense complement effector molecules such as C3a, C5a, C3b, iC3b, and C3d generated by DAMP-mediated activation of complement. Stress-induced signaling through PRRs on resident tissue cells and infiltrating leukocytes mediate tissue injury by attracting inflammatory cells and directly activate T-cells in the presence of foreign antigen and APCs that promote the donor-specific immune responses. Effector responses against donor antigen are also PRR signal dependent. The crosstalk between CR and TLR may alter the cellular response in a complex biological system [84].

**Figure 4:**
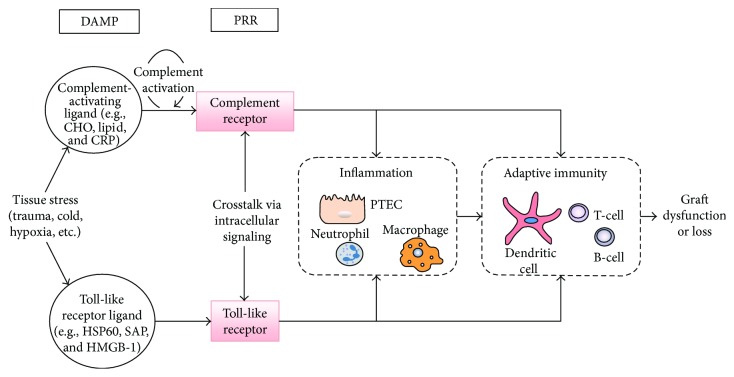
Pathway of injury mediated by innate immune receptors (adopted from [84]).

Neutrophils and macrophages express the cell surface receptors for C3a and C5a [87]. C3a elicits the release of prostaglandin E2 from macrophages, and C5a causes the release of histamine from mast cells. The activation of these cells induces endothelial cells to increase the expression of adhesion molecules such as endothelial cell selectin (E-selectin), vascular cell adhesion molecule 1 (VCAM1) and intercellular adhesion molecule-1 (ICAM1), cytokines, and chemokines such as interleukin-1*α* (IL-1*α*), IL-6, CCL-5, and CXC-chemokine ligand 8 (CXCL8). C3a and C5a also promote the mitogen-activated protein kinase signaling pathway, which is a key component of the signal transduction pathway that regulates the process of cell proliferation, cell differentiation, and cell death [88, 89]. The effector mechanisms are initiated by pattern recognition receptors and damage-associated molecular patterns as shown in Figure 4.

**Figure 5:**
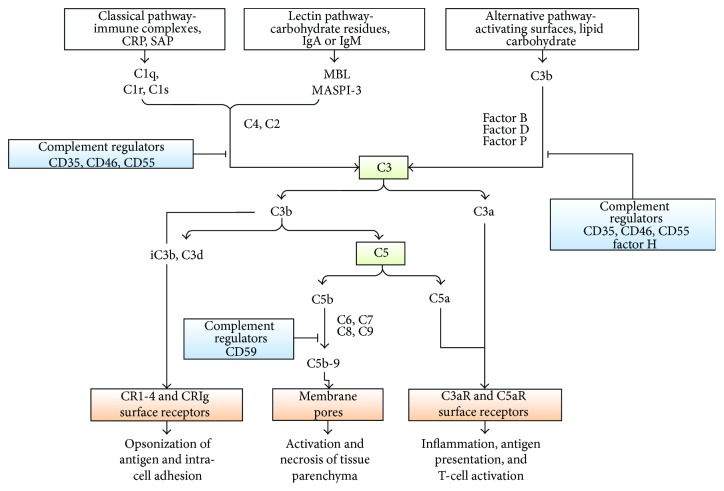
The complement cascades (adopted from [84]).

Complement is activated by classical, lectin, and alternative pathways (Figure 5). The classic complement activation pathway is relevant to antibody-mediated rejections [90]. The lectin pathway is initiated by mannose-binding proteins which bind to carbohydrate residues on the pathogenic surface or IgA and IgM molecules. The alternative pathway is triggered by direct binding of C3b to the activating surface. The classical pathway is triggered by the binding of C1q(C1q) to immune surveillance molecules that are attached to the target sequence (e.g., immunoglobulin), C-reactive protein (CRP), and serum amyloid protein (SAP) [84]. Activation of C1 (which is composed of C1q, C1r, and C1s) can be initiated by the interaction of the globular domains of C1q with IgG or IgM bound to antigen epitopes on the graft endothelium. The order of the C1q-binding potential of human IgG subclasses in decreasing capacity is IgG3, IgG1, IgG2, and IgG4. Conformational changes in C1q then allow cleavage of C1r, and activated C1r cleaves and activates C1s, which is the enzyme that activates C2 and C4. C4 is cleaved by C1s into the small fragment C4a and the large fragment C4b. C4d is a fragment of C4b, an activation product of the classic complement pathway. The effects of antibody on the endothelium of the renal allograft can be confirmed by C4d stains of renal biopsy which is a marker of complement activation-fixing circulating antibodies The interactions of antibodies with complement component begin the sequence that generates soluble peptides (C3a and C5a) and bound molecules (C4b and C3b) and culminates in the formation of the terminal complement components of the membrane attack complex [89].

All three pathways progress to form enzyme complexes that convert C3 and then C5 into active forms. This generates groups of complement effectors that mediate inflammation, antigen uptake, and B-cell stimulation. C5b is a multimeric complex that creates a pore in the target cell membrane and induces cell death [84]. C5b triggers the formation or assembly of membrane attack complex (MAC) composed of C5b–C9. This full activation of complement leads to graft rejection through cell leakage or lysis [87, 89]. The local necrosis and detachment of endothelial cells from the basement membrane are also indications for AMR [91]. Regulators of complement activation pathways are soluble (e.g., factor H) or membrane-associated, for example, CD35 (complement receptor 1 (CR1)), CD46 (membrane cofactor protein (MCP)), and CD55 (decay accelerating factor (DAF)). The regulators bind C3b (and C4b) and increase its decay or proteolysis from the C3 and C5 convertases of the classical and alternative pathways. Other regulators inhibit the formation of C5b–C9 (e.g., through binding of C3 by CD59) [84] (Figure 5).

**Figure 6:**
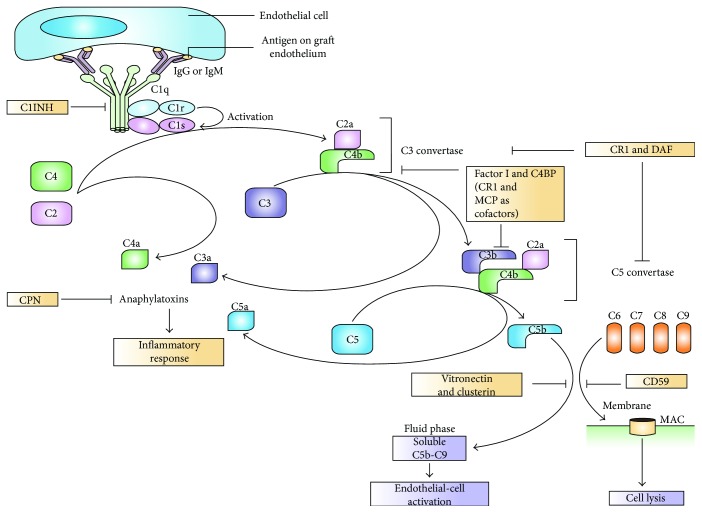
Classical pathway of complement activation by antigen-antibody (adopted from [89]).

Inhibitor proteins regulate complement activation, including C1 inhibitor (C1INH), carboxypeptidase N (CPN; which inactivates the anaphylatoxins C3a, C4a, and C5a), and factor I (which inactivates C3b and C4b, using C4b-binding protein (C4BP)). The membrane regulators complement receptor 1 (CR1), membrane cofactor protein (MCP), and decay-accelerating factor (DAF) regulate complement activation by functioning as cofactors for factor I-mediated cleavage of C3b and C4b (in the case of CR1 and MCP) or by accelerating the decay of C3 convertase and C5 convertase (in the case of CR1 and DAF). Fluid-phase activation causes C5b–C6–C7 complexes to bind vitronectin and clusterin, which are fluid-phase regulators of the terminal pathway. CD59 prevents the binding of C9 to C5b–C6–C7–C8 complexes in the terminal pathway. Many of the biological effects resulting from complement activation are mediated by cell surface receptors, such as the receptors for C1q, C3a, C5a, and iC3b (the inactivated form of C3b). Antibody-independent complement activation might also occur by the lectin and/or alternative pathways [89] (Figure 6).

**Figure 7:**
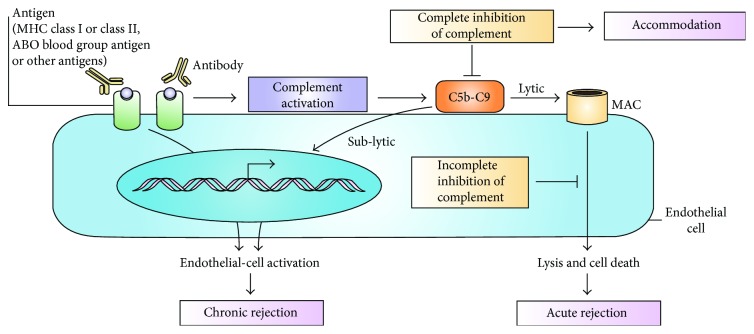
The three postulated outcomes of the binding of complement-fixing alloantibody to endothelial cells (adopted from [89]).

Donor-specific antibodies to HLA class I or II antigens can cause acute rejection such as by promoting coagulation and chronic rejections [90, 92, 93]. If complement activation is completely inhibited, the state of graft acceptance or resistance is known as accommodation [89] (Figure 7).

#### 2.1.3. Complement Components: C1q and C3

The complement system is composed of different subunits and functions as recognition (Clq, Clr, and Cls), activation (C4, C2, and C3), and attack (C5, C6, C7, C8, and C9) [94, 95]. C1q protein deficiencies result in the development of systemic lupus erythematosus, accumulation of autoantibodies, and apoptotic cells [96, 97]. Thus, serum C1q-circulating immune complexes could serve as a predictive diagnostic marker for renal flares in patients with lupus nephritis [98].

In humans, IgM and IgG bind to the multivalent C1q molecule and are capable of catalyzing the activation of C3 (an abundant protein and central component mediating all complement pathways). C3-triggering events may result in lyses of target cells by disrupting the plasma membrane [99]. C3 modulates both innate and adaptive immune responses, and its activation is involved in tissue damage [100]. A study reported that mice deficient in C3 are hematologically normal under steady-state conditions but displayed a significant delay in hematopoietic recovery from transplantation of wild-type mice [101]. Thus, complement inhibitors could be therapeutic options to intervene against transplantation rejection. Therefore, C3 represents a “hot spot” for complement-targeted therapy in the future [100].

Although complement activation is known to have a deleterious effect on organ transplantation, it has an important role in regulating immune responses such as immune complex clearance [99]. In the context of transplantation, the balance between C1q and C3 is found to be critical. A study reported that deficiency in C1q or C3 in female mice results in a rapid rejection of male skin grafts. It is because of the accumulation of T-cells which play a role in mediating inflammatory processes and graft rejection. Therefore, C1q may contribute in maintaining self-tolerance against foreign tissues [102]. However, the exact mechanisms for the failure of self-tolerance induction in C1q- and C3-deficient mice remain unexplored.

#### 2.1.4. Lymphocytes

The central problem in transplantation therapy is the immune response of T and B lymphocytes of the host against the donor antigens. T-cells have an ability to recognize genetically different MHC molecules. Usually, acute organ rejection is mediated by T-cells by either through T-cell-derived lymphokines or T-cell-mediated cell lysis [12]. T-cell-mediated reactions can take place through CD4 T-cells for HLA class II antigens or cytotoxic CD8 T-cells for HLA class I (A, B, or C) antigens [32]. The majority of B-cells require help from T-cells to initiate antibody production. Antibodies are widely recognized as the first causes of allograft failure [103]. Thus, T-cell-mediated rejection is a major determinant of inflammation in kidney allografts.

### 2.2. Antibody-Mediated Graft Rejection

Antibody-mediated rejection (AMR) is defined as allograft rejection caused by antibodies of the recipient directed against donor-specific HLA molecules and blood group antigens [104, 105]. Although the mechanism by which HLA I antibodies promote inflammation and proliferation has been revealed by experimental models, the pathogenesis of HLA II antibodies is less defined. Antibodies to HLA II frequently accompany chronic rejection in renal transplants [71]. AMR has been recognized as the leading cause of graft loss after kidney transplant if there is a donor-host antigenic disparity. Antibodies can be produced against epitopes of the antigen that differ from self by as little as one amino acid [19]. Preexisting antibodies or the development of de novo antibodies after transplantation has become a biomarker for AMR graft loss [72, 106]. HLA antibodies are risk factors for hyperacute, acute, and chronic allograft rejections [2].

The specificity of HLA antibodies can be determined using single-antigen luminex beads that consist of fluorescent microbeads conjugated to single recombinant HLA class I and class II molecules. Complement-fixing ability would be assessed by the binding of C1q to HLA antibodies present in the serum. In several studies, C1q-positive DSA had associated with antibody-mediated rejection in renal transplantation compared with antibodies identified only by IgG [71, 107]. Complement-fixing ability is relevant to hyperacute and acute rejections. Hyperacute rejection is predominantly complement-mediated severe allograft injury occurring within hours of transplantation. It is caused by high titer of preexisting HLA or non-HLA antibodies in presensitized patients. But the incidence of hyperacute rejection is reduced due to improved DSA detection methods and desensitization protocols [71]. Patients with class I DSA had worse graft survival than class II. C1q testing in pretransplant sera with DSA was unable to predict acute antibody-mediated rejection or early graft loss. Despite nonfixing complement *in vitro*, pretransplant C1q-negative DSA I can mediate rejection and graft loss [108].

The mode of action of antibodies in transplant rejection can be mediated by damage of endothelial cells due to the activation of complement-mediated cytotoxicity, by induction of antibody-dependent cytotoxicity (ADCC), through intensification of inflammatory reactions by the release of complement components (C3a, C5a), or by immune complexes (activation of clotting system) [42, 90]. The main antigenic targets of AMR are HLA molecules (class I and class II) [9] and ABO blood group antigens [89]. Acute AMR remains a significant challenge of kidney transplantations occurring from 20 to 50% [33], whereas chronic rejection accounts for 50 to 80% [109].

Antibodies directed against donor antigen can cause different types of rejection that can vary in acuity and severity. The main types of graft rejection are hyperacute, acute, and chronic rejections [110]. Hyperacute rejection refers to previous sensitizations leading to preformed antibody [111] that causes immediate (minutes or hours) vascular injury via ADCC. Acute rejection involves cellular infiltrates of both CD4+ T-cells and CD8+ T-cells. Acute rejection occurs within days (sometimes), months (usually), and years later when immunosuppressive therapy is discontinued. Chronic rejection usually occurred from three months to years. During chronic rejection, both T-cells and antibodies are involved. The development of de novo antibodies after transplantation is associated with chronic AMR [71, 111]. Clinically, chronic rejection remains the major unresolved problem in transplantation [112].

Antibodies to donor HLA class I or II antigens are present in 88 to 95% of the patients who have C4d deposition [113, 114]. Antibodies to donor ABO antigens show a similar association [115]. However, not all patients with AMR have anti-HLA antibodies, indicating that other non-HLA factors such as MHC class I-related chain A (MICA) antigens are involved in acute or chronic graft damage. MICA antigens can be found in fibroblasts, endothelial cells, dendritic cells, and many tumors. MICA antigens are structurally similar to MHC class I proteins, closely linked to HLA-B and C loci [91–93].

**Figure 8:**
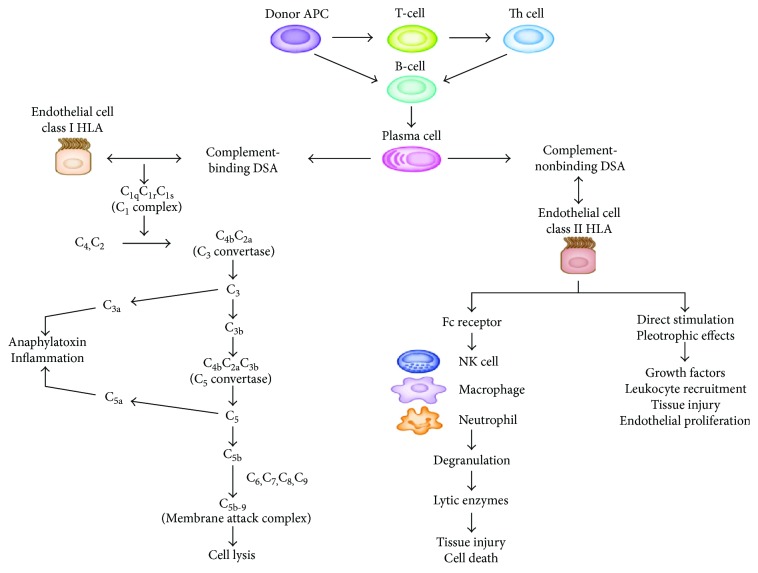
Mechanisms of pathogenesis of donor-specific antibodies in antibody-mediated rejections (adopted from [42]).

Antibodies produced by plasma cells activate the complement system involved in AMR [93]. In a sensitized transplant recipient, memory B lymphocytes in bone marrow, spleen, and lymph node results in the formation of antibody-secreting plasma cells that produce high levels of DSA [33]. Alloantibodies recognize antigenic epitopes displayed by HLA molecules on the transplanted allograft through complement-dependent and independent pathways [89]. This activates the complement system to generate inflammatory split products and engagement of Fc gamma receptors (Fc*γ*R) on natural killer (NK) cells, monocytes, and neutrophils [19, 116]. Complement-binding DSA target class 1 HLA on endothelial cells, activate the classic complement cascade, and deliver complement-dependent cytotoxicity in acute antibody-mediated rejection. Complement-nonbinding DSA recruit innate immune cells (NK cells, macrophages, and neutrophils) through Fc receptors and lead to antibody-dependent cellular toxicity. In addition, complement-nonbinding DSA have direct stimulation and pleiotropic effects that cause tissue injury, cellular recruitment, and endothelial proliferation. The latter two mechanisms play an important role in acute antibody-mediated rejection with a negative C4d deposit in peritubular capillaries as well as chronic antibody-mediated rejections [42] (Figure 8).

Antibodies of the IgG isotypes and possibly IgM isotypes are clinically relevant for transplantation [117]. However, preexisting IgG isotypes are considered the main risk factors for AMR [10]. IgG1 and IgG3 are the most efficient activators of the complement system [92]. Endothelium of donor graft peritubular and glomerular capillaries displays MHC molecules which are the target for antibody production. Once the endothelium is damaged by an antibody, factors such as P-selectins are released as an inflammatory response. Leukocytes adhere to glomeruli or to dilated peritubular capillaries via cytokines (IL-1*α*, IL-8) and chemokine ligand 2 allowing complement activation [91]. Although single-antigen bead assay is developed to detect donor-specific antibodies, the definition of antibody attributes that are associated with AMR pathology remains unclear.

There are three major effector functions carried out by antibodies. First, bivalent IgG can dimerize or crosslink its target upon binding to HLA and triggers downstream activation of the target cells. Second, antibodies can activate the classical complement cascade through binding to Fc-fragment which triggers the production of potent anaphylatoxins, chemoattractants, opsonins, and cell-damaging factors. Thirdly, HLA IgG bound to target cells can engage Fc receptors on myeloid and lymphoid cells to employ a host Fc receptor-mediated effector functions, including ADCC and antibody-dependent phagocytosis [12]. The destructive power of alloantibodies of the recipients directed against HLA class I and II molecules varies [112], depending on the level of antibody [33], immunoglobulin isotype, target antigen, and the type of organ transplanted [117, 118]. High-titered pretransplant antibodies directed against HLA class I antigens can cause catastrophic hyperacute rejection [19].

The current kidney allocation algorithms used by most transplant societies including the USA consider only antigens in HLA-A, B, and DR loci [119]. HLA-DR molecules are considered to be relevant for graft rejection, because its *β*-chain is polymorphic and contributes to differences for HLA-DR alleles, but the *α*-chain is found virtually nonpolymorphic [18].

#### 2.2.1. DQ/DP Antibodies and Acute Rejection

Although HLA-DQ/DP gene regions possess polymorphic chains (the *α*-chain and the *β*-chain), the effects of their mismatches on transplant outcome have been less certain until recently. Thus, HLA-DQ/DP antigens expressed on the cell surface promote peptide binding to class II molecules [2] and need to be considered for transplantation therapy [120–122]. The reason why the effects of HLA-DQ matching have been underemphasized in the past years was the assumption that HLA-DQ matching was closely parallel to HLA-DR matching because of linkages in the proximity of the two genes on chromosome 6. In addition, the identification of HLA-DQ or DP molecules has relied on DNA sequencing than the common HLA methods [119]. On the other hand, HLA-DQ and HLA-DP mismatches do not appear to be important for transplantation [3].

An increasing recognition of either preformed or de novo anti-HLA-DQ-DSA and their role in acute and chronic rejection suggests the need to assess the risk of transplantation at the epitope levels [119]. A study reported that HLA-DQ antibodies are the most commonly developed de novo DSA among posttransplants. Thus, there is a positive correlation between the presence of donor-specific HLA-DQ antibodies and an increased risk of transplant rejection [120–122]. The effect of anti-HLA-DQ-DSA relates to the presence of the high number of polymorphic epitopes expressed on the *α*- and *β*-chains [123]. Reports [120] demonstrated that HLA-DQ mismatches are associated with acute rejection independent of HLA-A, B, and DR mismatches. Acute graft rejection was significantly worse when HLA-DQ antibodies were combined with non-DQ antibodies compared with HLA-DQ alone or no antibodies. There was a significant association between HLA-DQ mismatches and acute rejection among patients who had received HLA-DR mismatched kidneys [119, 124]. This implies the occurrence of an enhanced immunogenicity of HLA-DQ loci and production of de novo anti-HLA-DQ-DSA associated with the existence of mismatched HLA-DR [121]. Moreover, a study revealed that C1q more likely has DQ-DSA specificity and is associated with 30% reduction of graft survival to reach the 5th year [125, 126]. Therefore, HLA-DQ antigens of the donor and recipient should be taken into account for kidney transplantations [127, 128].

Similarly, there are conflicting reports on the clinical relevance of antibodies directed against HLA-DP antigens. The presence or absence of HLA-DP antibodies did not affect graft survival among pretransplant and posttransplant patients [129]. In contrast, HLA DP have been considered to be less immunogenic than HLA- A, -B, DR, and DQ molecules [130]. Reports revealed that patients with high levels of pretransplant donor-specific HLA-DP antibodies developed acute rejection in the absence of other donor-specific HLA alloantibodies [131]. Therefore, it is important to consider HLA-DP epitope mismatches to monitor its impact on graft rejection [132].

## 3. Antigen Processing Pathways

MHC-I and II molecules show strong preferential restrictions for the origin of proteins they sample for presentation. The MHC-I antigen presentation pathway is an event of an inside-out (endogenous antigens) process by which protein fragments synthesized by the cell are delivered to MHC-I molecule. Peptides derived from proteins in the cytosol are degraded by multiproteolytic enzymes and transported to the endoplasmic reticulum with the help of an intrinsic membrane transporter, then, displayed to T-cells through TCR recognition. MHC class I glycoproteins recognize antigens derived from infecting bacteria, viruses, intracellular parasites, or self-molecules. In contrast, the MHC-II antigen presentation pathway is visualized as an outside-in (Exogenous antigens) process in which ingested proteins are degraded by enzymes in the endosomal-lysosomal system and delivered to MHC-II molecules in the degradative compartment. MHC class II molecules bind peptides (or nonpeptides) and display at the cell surface for recognition by antigen-presenting cells [16].

There are three pathways by which HLA are recognized by the recipients. These include the direct, semidirect, and indirect pathways. The direct pathway of alloantigen recognition is unique to transplantation. Allogeneic MHC class I and II antigens are presented to recipient CD4+ and CD8+ T-cells by donor APCs [19, 133]. In other words, alloreactive T-cells recognize intact donor MHC molecules on APCs that are “passengers” in the transplanted tissues. DCs of the donor and the recipient can provide activation signals to recipient T-cells [134]. The direct recognition of alloantigens may give rise to cytotoxic CD8+ and CD4+ cells, as well as to Th1 or Th2 cytokines that will trigger delayed-type hypersensitivity (DTH) and eosinophil rejection, respectively [81]. The semidirect pathway involves host APCs such as DCs which present intact donor antigen taken up as a membrane patch. Processed donor peptide complexes are presented into the recipient's CD8+ T-cells (class I) and helper CD4+ T-cells (class II). In the indirect pathway, host APCs present peptides or antigens derived from the donor MHC molecules to recipient T helper cells and cytotoxic T lymphocytes [133]. Alloantibodies are generated when alloreactive B-cells interact with CD4+ T-cells [19, 135]. The repertoire of T-cells involved in the indirect recognition of allo-MHC peptides is responsible for alloantibody synthesis, and these T-cells may also lead to Th1/DTH or Th2/eosinophil rejection [81].

CD4+ T-cells can differentiate into two different subsets whose functional properties are characterized by the cytokines they secrete. T helper (Th)1 cells produce interferon- (IFN-) *γ* and IL-2, which results in the activation of CD8+ cytotoxicity, macrophage-dependent delayed-type hypersensitivity, and the synthesis of complement-fixing antibody. In addition, Th1 cells may become cytotoxic by the expression of Fas ligand on their surface. Th2 cells secrete IL-4, IL-5, IL-9, IL-10, and IL-13. This mainly triggers eosinophil activation, a process that can by itself mediate graft rejection [81]. IFN-*γ* is the principal lymphokine from T-cells that activates macrophages to become more cytotoxic and enhances MHC antigen expression in many cells. Tumor necrosis factor alpha (TNF-*α*) is a cell signaling cytokine involved in systemic inflammation and results in an acute reaction. The cytokines from macrophages such as IL-l (activation of T-cells, endothelial procoagulant induction) and TNF-*α* (cytotoxicity inductions) are involved in chronic rejections [12].

## 4. Therapeutics to Avoid Graft Rejection

If a transplant candidate is already sensitized, graft rejection can be minimized through perfect crossmatch for HLA typing (class I and II) between donors and recipients [48]. After transplantation, screening for the presence of de novo alloantibodies and monitoring adherence to immunosuppression are obligatory for the management of AMR [69, 106]. The recipient body will attack the new kidney transplant considering as nonself. The immunosuppressant drugs suppress your body's ability to do this. After transplantation, patients need to take immunosuppressive drugs continuously to ensure that the immune system is adequately suppressed allowing the graft to survive [7]. Although HLA matching minimizes antigen disparities, there are still “minor histocompatibility antigens” which affect the transplantation outcome.

The principle of desensitization for ABO and HLA incompatible transplants is the removal of antibody, reduction in antibody production, and augmented immunosuppression supplement. The most common treatment strategies or desensitizations are the reduction of antibody titer levels of the recipient which render transplantation safety [22, 89]. Identifying the molecular pathways that trigger tissue injury and signal transduction facilitates the identification of targets for the immunosuppressive treatment [103].

Current treatment strategies in AMR are centered on the depletion of both naive and memory B-cells, downregulation of alloantibody production by plasma cells, blockade and elimination of alloantibodies, and modulation of alloantibody-induced injury. In the absence of an effective plasma cell depletion agent, splenectomy is the most efficient method for the reduction of the plasma cell mass [105]. All kidney allograft recipients are given immunosuppressants to prevent rejection [22]. Almost everyone who has a transplant must take these drugs every day as directed. Transplant recipients are maintained on an immunosuppression regimen that includes 1–3 drugs [27]. However, such prolonged treatment results in infections [2]. Immunosuppressive drugs which are used in clinical transplantation are outlined in Table 1.

## 5. Future Directions

Prevention of graft rejection remains a common problem in transplantation therapy. The major obstacles for a successful kidney transplantation are graft rejection, adverse effects of immunosuppressive drugs [134], and lack of reservoir organs for transplantation [1]. In addition, sensitization to HLA represents a barrier to transplantation for patients who develop donor-specific anti-HLA antibodies (DSA) as a result of pregnancy, blood transfusions, or previous transplants [136]. This results in prolonged waiting times for transplantation [33], and if transplanted, these patients are at higher risk of acute and chronic rejection [134, 137]. Thus, the detection of humoral sensitization before renal transplantation is important for the selection of the most suitable donor and to identify patients with high risk of rejection [117]. When a patient is already sensitized, precise characterization of alloantibodies and exact HLA typing at the allele level are mandatory at the time of transplantation [69]. Moreover, the knowledge of HLA sensitizations and identification of anti-HLA antibodies among potential renal recipients are essential to control graft loss [138]. The approaches to enhance graft survival are gaining acceptance in human tissues and organ transplantation. A better understanding of the cellular and molecular mechanisms that underline the immunological response to transplanted organ led to the discovery of new immunosuppressive agents [2].

The ultimate goal of renal transplantation is to generate donor-specific immunologic tolerance (acceptance of allograft). New immunosuppressive drugs without having long-term overall immunosuppression are required [22, 139]. New therapeutic strategies targeting TLRs, NK cells, complement such as humanized anti-C5 antibody, and monocytes or DCs of the innate immune system will be necessary to prevent antibody-mediated rejection and eventually achieve long-term tolerance to human allograft recipients [38]. Similarly, inhibitors of the complement system may be potential targets of future therapy. Complement antagonists could prevent the acute pathological effects of complement activation such as the blockage of C5a, and the MAC formation prevents acute rejection [89]. The prevention of antibody-mediated endothelial cell injury through complement blockage and the depletion of DSA secreting plasma cells from the bone marrow using proteasome inhibitions are potential areas of further studies [33]. The management of both acute and chronic rejections suffers from the lack of effective antiplasma cell agents that would allow for faster elimination of antibody production [105]. Thus, treatment targets that lower the production of DSA are important for allograft survival [33, 105]. Future drugs targeting both type 1 (Th1 cells) and type 2 (Th2 cells) effector mechanisms are required. In addition, tolerance induction through blockade of costimulatory molecules could be a potential area of future research to investigate immunosuppressive drugs.

Notch signaling is a highly conserved cell-to-cell communication pathway triggered by Notch ligand-receptor interactions between adjacent cells. It plays an important role during T-cell development, and it is the central mediator of T-cell alloreactivity. Short-term inhibition of individual Notch ligands in the peritransplant period had long-lasting protective effects. Blockade of delta-like Notch ligands dampened both cellular and humoral rejections in the heart allograft model. Therefore, it has been proposed that targeting elements of the Notch pathway could provide a new therapeutic approach to prevent allograft rejection by damping proinflammatory cytokine production by Teff and enhancing both T regulatory (Treg) functions [140]. In animal models, Tregs can prevent transplant rejection. Therefore, administration of Tregs to transplanted patients is a potential treatment target to induce graft tolerance and allow a reduction in doses of immunosuppressive drugs [27]. Memory B-cells are heterogeneous but have cell surface markers (CD24, CD27, CD43, and CD79) that are potential therapeutic targets [89].

### 5.1. Epitopes in HLA Matching

Currently, serological HLA matching has been used as the standard algorithm for solid organ allocation. However, not all antibody mismatches are pathogenic [141]. Antibodies do not recognize whole HLA molecules, but only polymorphic residues on HLA surfaces. Such HLA segment sequences targeted by an antibody would consist of 15 to 25 amino acids termed as an epitope [142]. In its first version, each HLA having polymorphic amino acid sequences with an antibody-binding position is known as triplets, which are considered the key elements of epitopes [143]. Generally, epitopes may be private or public antigens [144]. Thus, HLA antibodies could be unique to individual antigens due to private epitopes or result in cross-reactivity in HLA testing because of public epitopes shared by multiple antigens. Thus, a better understanding of HLA epitopes and the interpretation of antibody reactivity pattern is required in transplantation therapy [145].

The determination of antibody strength (antibody titer or mean fluorescence intensity) and the ability to fix the complement are necessary for permissible transplant matching. The measure of mean fluorescence intensity (MFI) of serum antibody concentration, strength, and potential pathogenicity is not a perfect match. However, MFI considerations would be most applicable for patients without sensitization history [141]. Epitope matching reduces the likelihood of developing de novo HLA antibodies and lowers the risk of graft rejection [146]. Although there is a difficulty in distinguishing between positive, weakly positive, and negative reactions, HLA epitope antibody verification is currently performed using luminex bead assays [147].

### 5.2. HLAMatchmaker

HLAMatchmaker is a computer algorithm developed to evaluate donor-receptor compatibility of polymorphic amino acids (eplets) present in HLA molecules. It is a promising tool in predicting anti-HLA antibodies with better sensitivity than the former HLA matching methods [148]. It is used to analyze serum antibody reactivity patterns of sensitized patients and their potential donors with acceptable matches. The eplet may provide a more accurate evaluation of HLA compatibility. HLAMatchmaker works based on the following two principles: first, each HLA is represented by different chains of epitopes structurally defined as potential immunogenic agents capable of inducing specific antibody production; and second, patients cannot produce antibodies against epitopes present on their own HLA molecules [143]. HLAMatchmaker has the ability to determine epitope specificities of highly sensitized individuals comparing eplet mismatches between a donor and a recipient [149].

Donor-specific HLA antibody formation after kidney transplantation is associated with donor-derived HLA epitopes presented by recipient HLA class II (predicted indirectly recognizable HLA epitopes presented by HLA class II, PIRCHEII). Each PIRCHE-II represents a peptide with potential immunogenicity, but with an unknown degree of immunogenicity. PIRCHE-II immunogenicity may differ per peptide due to preferential processing and/or binding to HLA. Immunogenic HLA contain higher PIRCHE-II numbers than nonimmunogenic HLA. For instance, during pregnancy, the number of PIRCHE-II is related to the formation of child-specific HLA antibodies. Therefore, the role of PIRCHE-II in antibody formation outside the transplantation setting suggests the need for defining the immunogenicity of individual PIRCHE-II, which gives more insight into the clinical relevance of each individual PIRCHE-II [150].

## Figures and Tables

**Table 1:** Common immunosuppressive agents.

Number	Drugs	Mechanism of action	Effect	Reference(s)
1	Mycophenolate sodium, tacrolimus, and azathioprine	Inhibits signals transmitted by IL-2 binding to IL-2R (antiproliferating effect)	Blocks T-cell activation, decreases both cell-mediated and humoral immunities	[27, 133]
2	Glucocorticosteroids: prednisone	Anti-inflammatory	Decreases circulating T-cells and inflammatory cytokines	[27]
3	Polyclonal antithymocyte globulin (ATG) or antilymphocyte globulin (ALG)	Leucocyte depletion/depleting antibodies. Eliminates CD4+ T-cell and B-cell interaction causing B-cell toxicity/apoptosis	Modulation of alloantibody production	[27, 91]
4	Mycophenolate mofetil	Inhibits inosine monophosphate dehydrogenase (IMPDH), inhibits DNA synthesis and protein glycosylation, suppresses expression of CD25, 71, 154, 28	Decreases proliferation of B and T-cells	[27, 133]
5	Anti-CD3 monoclonal antibody	T-cell activation, opsonization, and depletion of antibodies		[27, 133]
6	Tacrolimus, cyclosporine A	Inhibits interleukin- (IL-) 2 production by T-cell calcineurin antagonist, gene transcription, calcineurin inhibitors; causes decrease in gene expression	Decreases both cell-mediated and humoral immunities	[27, 133]
7	Anti-CD 20 monoclonal antibody (chimaeric)	Targets B-cells, depletes B-cell aggregates within allografts	B-cell depletion	[27, 33, 69]
8	Anti-CD 25 monoclonal antibodies (IL-2R chain)	Inhibits IL-2 function		[27]
9	Plasmapheresis, mycophenolic acid	Reduction of antibody titers		[89]
10	Intravenous immunoglobulin (IVIG)	Reduces CD19, CD20, and CD40 expression by B-cells	Blocks the binding of donor-reactive antibodies to target Fc receptors. Regulation of T and B lymphocytes	[33, 89, 135]
11	Rituximab	B binds with CD20 antibody, inhibits B-cell proliferation, decreases the concentration of antibodies. Antibody-dependent cellular cytotoxicity, direct signaling, and antibody-mediated cytotoxicity	Decreases the population of CD20 B-cells.	[33, 77, 133]
12	Plasmapheresis	Removal of DSA in circulation (elimination of DSA)	Reducing the antibody load	[91, 110]
13	Immunoadsorption	Treatment of multiple plasma volumes		[69]
14	OKT3 (murine) anti-CD3/TCR monoclonal antibodies	TCR comodulates with CD3		[90]
15	Eculizumab (humanized monoclonal antibody)	Binds to the C5 protein with high affinity, thereby inhibiting conversion of C5 to C5b.	Preventing formation of the membrane attack complex (C5–9)	[110]
